# The Role of Extracellular Vesicles in Vein Graft Disease

**DOI:** 10.3390/cells15100916

**Published:** 2026-05-17

**Authors:** Georgia R. Layton, Riyaz Somani, Giovanni Mariscalco, Farooq Donoo, G. André Ng, Ibrahim Antoun, Mustafa Zakkar

**Affiliations:** 1 College of Life Sciences, University of Leicester, Glenfield Hospital, Leicester LE3 9QP, UK; 2 Department of Cardiac Surgery, University Hospitals of Leicester NHS Trust, Glenfield Hospital, Leicester LE3 9QP, UK; 3 Leicester British Heart Foundation Centre of Research Excellence, Glenfield Hospital, Groby Road, Leicester LE3 9QP, UK; 4 Department of Cardiology, University Hospitals of Leicester NHS Trust, Glenfield Hospital, Leicester LE3 9QP, UK; 5 Department of Anaesthetics, University Hospitals of Leicester NHS Trust, Glenfield Hospital, Leicester LE3 9QP, UK; 6 National Institute for Health Research, Leicester Research Biomedical Centre, Leicester LE5 4PW, UK

**Keywords:** extracellular vesicles, vein graft disease, intimal hyperplasia, microRNA, KCa3.1, vascular smooth muscle cells

## Abstract

Coronary artery bypass grafting (CABG) using the autologous saphenous vein (SV) remains widely performed for obstructive atherosclerosis; however, vein graft disease drives recurrent ischaemia through early thrombosis and progressive intimal hyperplasia, and accelerated atherosclerosis developing within the grafts. Extracellular vesicles (EVs) are membrane-bound particles that transfer proteins, lipids, and microRNAs between cells. They modulate endothelial dysfunction, vascular smooth muscle cell phenotypic switching, inflammation, and coagulation, which are core processes in vein graft remodelling. Arterialisation exposes the vein to abrupt rises in shear stress, cyclic stretch, and intraluminal pressure. These forces increase EV release and reshape EV cargo in experimental systems, suggesting a potential mechanism for amplifying early graft injury which warrants direct investigation in vein tissue. This review synthesises current evidence for cell-specific EV contributions from ECs, vascular smooth muscle cells, platelets, and macrophages, and appraises EV-associated microRNAs with biomarker potential relevant to graft failure pathways. We also review therapeutic strategies that may modulate EV signalling including antiplatelet therapy, statins, KCa3.1 inhibition, and pro-reparative mesenchymal stromal cell-derived EVs. No published clinical studies evaluate EV-based biomarkers specifically for saphenous vein graft patency, and none prospectively predict saphenous graft failure. CABG provides a well-defined time zero event that enables longitudinal sampling and risk stratification. Prospective studies linking EV phenotypes and miRNA signatures to imaging-defined graft outcomes are needed to support clinical translation.

## 1. Introduction

Cardiovascular disease remains the leading cause of mortality globally, with coronary artery disease (CAD) being one of its most prevalent forms [1]. Coronary artery bypass grafting (CABG) is one of the most commonly performed cardiac surgical procedures for multivessel CAD, and autologous saphenous vein (SV) grafts are used in the majority of cases [2,3]. Despite advances in surgical technique and peri-operative care, vein graft disease (VGD) continues to compromise long-term outcomes. Approximately 10–20% of SV grafts occlude within the first year, and up to 60% develop significant stenosis within 10 years [3].

Vascular homeostasis depends on continuous communication among endothelial cells (ECs), vascular smooth muscle cells (VSMCs), platelets, and immune cells. These cells coordinate vascular tone, vessel repair, and remodelling through paracrine signalling [4]. The pathological processes in vein grafts differ from those in native arteries. They are initiated by the abrupt mechanical stress of arterialisation and by the surgical injury sustained during vein harvest, and they progress on a compressed timeline, in which intimal hyperplasia (IH) precedes any lipid-driven plaque formation [5]. During harvest and subsequent arterialisation, the SV undergoes a series of adaptive and maladaptive changes, including endothelial denudation, VSMC proliferation, extracellular matrix remodelling, and inflammatory cell infiltration, collectively driving IH, which can progress to narrowing or occlusion of the venous lumen. When luminal obstruction of a vein graft results in myocardial hypoperfusion, it can be termed VGD or failure [5].

Extracellular vesicles (EVs), containing proteins, lipids, and nucleic acids, are membrane-bound particles released by cells and function as intercellular messengers, with both diagnostic and therapeutic potential [6]. Exosomes (30–120 nm) originate from multivesicular bodies through ESCRT (endosomal sorting complexes required for transport)-dependent and -independent pathways, whilst microvesicles (150–1000 nm) form the outward budding of the plasma membrane, triggered by calcium-dependent cytoskeletal reorganisation [6,7]. Cells also release apoptotic bodies and non-vesicular extracellular particles, although their biogenesis is less well characterised. Given the overlap in EV subtypes by size, density, and molecular composition, and the challenges of separating them during purification, throughout this review we will not specify EV subtypes and will collectively refer to these molecules as EVs, as recommended by current guidelines [8].

We retain subtype-specific terminology in two circumstances. Where the original study purified and characterised a defined subpopulation, we report the finding using the authors’ designation. Where the original study did not resolve the subtype, we use the collective term EVs and avoid attributing the mechanism to a specific biogenesis pathway. This approach follows the intent of the MISEV2023 guidelines [8], which permit collective terminology where subtype purification is not demonstrated but expect authors to discuss subtype implications when biogenesis-specific biology affects interpretation. We apply that distinction explicitly in the therapeutic section, where the targets of individual agents are subtype-restricted.

This review synthesises current evidence on EV biology in the context of SV graft pathology; we assess the emerging potential of EV-associated miRNAs as diagnostic biomarkers and review therapeutic strategies for EV modulation. The absence of vein graft-specific data is a recurrent limitation which currently prevents clinical translation and is addressed directly at the end of this review. Throughout this review we apply an explicit hierarchy of evidence where the strongest tier is data directly from human SVs, of which very few studies exist. The second tier is data from venous cell systems and venous interposition graft models from non-human animals. The third tier is data comprising arterial cells and injury models including human aortic and smooth muscle cells, human umbilical venous ECs used as a generic EC model, and mouse aortic models. The fourth tier is vascular cell systems used to characterise generic EV biology. Most cited evidence within this review sits within the third and fourth tiers only. We indicate the tier of study at its first mention and reserve any mechanistic claims for findings if supported by the upper two tiers of data.

A prior review [9] addressed EVs in neointima formation after vascular injury more broadly, drawing largely on arterial injury models. The present review differs from this prior review. We focus specifically on SV graft biology, placing mechanotransduction at the implantation of the vein into a coronary artery system at the centre of the conceptual framework and we provide an explicit appraisal of the vein graft evidence gap and its implications for clinical translation.

### Venous and Arterial Vascular Cell Biology

SV and coronary artery cells differ in ways directly relevant to EV biology [5,10]. Venous smooth muscle cells switch more readily to a synthetic, proliferative state in response to mechanical injury compared to their arterial counterparts, as reviewed in the context of vein graft physiology [5,11]. Venous ECs are exposed to lower baseline shear stress than the arterial endothelium, and the shear-responsive transcription factor KLF2, which regulates endothelial nitric oxide synthase and drives the packaging of miR-143 and miR-145 into endothelial EVs, has been characterised almost exclusively in arterial endothelial models [12,13,14]. Whether the same packaging machinery operates in the venous endothelium exposed to a sudden increase in shear stress after implantation into the coronary arterial circulation, is unknown. These differences are impactful for this review because the EV cargo released by an arterialised vein graft has not been directly characterised, and existing vein graft models have examined downstream signalling rather than EV composition itself [11,15]. Secondly, therapeutic strategies that act on EV biogenesis pathways which have been defined in arterial cells, such as KCa3.1-mediated exosome secretion, may show different efficacies in venous tissues [16,17,18]. Additionally, biomarker validation cannot rely on the translation of cell-of-origin signatures from arterial models without confirmation in both venous endothelial and smooth muscle cells directly. This limitation is illustrated by the only CABG cohort study linking circulating EVs to graft patency, which did not separate data from arterial and venous conduits [19]. This review should therefore be read with these limitations in mind.

## 2. Mechanotransduction and EV Release upon Vein Graft Arterialisation

**Figure 1:**
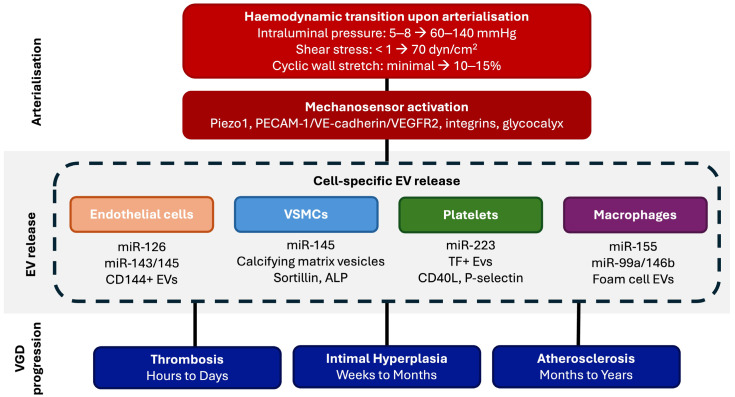
A proposed model of mechanotransduction induced extracellular vesicle release and how this could impact vein graft disease progression. The causal arrows linking EV release to disease outcomes are hypothesised from arterial evidence and have not been validated in human vein graft studies. Abbreviations: ALP, alkaline phosphatase; EV, extracellular vesicle; TF, tissue factor; VSMC, vascular smooth muscle cell.

SV grafts undergo a dramatic haemodynamic transition when implanted into the coronary circulation, and their adaptation to this is a process known as “arterialisation” [20]. Intraluminal pressure increases more than twenty-fold from its usual 5–8 mmHg to 60–140 mmHg, shear stress rises from <1 dyn/cm^2^ to 70 dyn/cm^2^, and cyclic wall stretch escalates from minimal to 10–15% [21]. These changes simultaneously activate mechanosensitive signalling cascades in both ECs and VSMCs [21]. The effects of these forces on EVs have not been evaluated in human SVs. However, evidence from other cell types demonstrates that changes in these forces significantly alter EV biogenesis, EV cargo loading, and EV release kinetics (Figure 1). It is therefore reasonable to expect that similar changes in these forces would result in similar effects within a grafted vein. The abrupt transition the vein is exposed to when moved from its low-pressure venous environment to a high-pressure arterial one represents a unique pathological stimulus that distinguishes VGD from native vessel atherosclerosis, and it is this mechanical stress that likely dramatically amplifies EV production across multiple experimental models [20]. Recent work has demonstrated that shear stress magnitude regulates not only EV release but also EV uptake. Coly et al. showed that high shear stress produces approximately 50 times more large EVs and five times more small EVs than low shear stress from ECs [22]. Low-shear-stress-derived EVs are preferentially taken up by recipient ECs via MCAM and PECAM-1 adhesion molecules, carrying a proteome enriched in mitochondrial and adhesion proteins [22]. This provides a direct mechanistic link between the disturbed flow environment of vein grafts and pathological EV-endothelial interactions.

Qin et al. [23] used red blood cell-derived EVs to study the effect of shear stress magnitude on EV uptake by ECs, both in vitro and in vivo in atherosclerosis models. They found that low shear stress (5 dyn/cm^2^) and oscillatory shear stress increased EC uptake of EVs by up to 189-fold compared with high shear stress (25 dyn/cm^2^), a mechanism driven by oxidative stress. In zebrafish, EVs preferentially accumulated where flow velocity was lowest, directly demonstrating that venous low-flow environments promote EV-endothelial interactions. The low and disturbed shear stress patterns seen in vein grafts would therefore favour enhanced EV uptake by ECs, potentially accelerating VGD. Similarly, Guo et al. [24] seeded dental pulp stem cells (DPSCs), adipose-derived mesenchymal stromal cells (MSCs), and skeletal muscle cells onto 3D scaffolds within bioreactors and applied either direct flow stimulation (0.5–1.0 mL/min, generating wall shear stress of 0.5–5 dyn/cm^2^) or cyclic stretch (25% strain at 1 Hz for 48 h). Flow-stimulated DPSCs produced up to 37-fold more EVs than static controls, while cyclic stretch increased skeletal muscle cell EV secretion 11-fold, all mediated through YAP mechanosensitivity, as confirmed by verteporfin inhibition experiments. These mechanical forces mirror those encountered by SV walls upon arterial implantation. Hou et al. extended these findings by demonstrating that ECs exposed to disturbed flow release EVs that polarise macrophages from an anti-inflammatory to a pro-inflammatory phenotype via the mitogen-activated protein kinase (MAPK) pathway, accelerating atherosclerosis in vivo [25]. Inhibition of MAPK signalling blocked this pathological EV release [25]. This connects the mechanotransduction evidence above directly to the macrophage-derived EV biology discussed later.

Hergenreider et al. [12] used human umbilical vein endothelial cells (HUVECs) to demonstrate that atheroprotective laminar shear stress (20 dyn/cm^2^) activates KLF2, which enriches endothelial EVs with miR-143/145 for transfer to co-cultured VSMCs (arterial origin), reducing their proliferative capacity. The EVs also reduced atherosclerotic lesion formation in the aortas of ApoE-/- mice. In contrast, disturbed flow fails to activate this protective mechanism and instead promotes pro-inflammatory EV cargo. This distinction in EV cargo between laminar and disturbed flow is particularly relevant to vein grafts, where flow patterns are often turbulent at anastomotic sites and within regions of geometric mismatch between a graft and the native vessel size. Recent work by Yao et al. [15] used a rabbit jugular vein-to-carotid artery interposition graft model to show that higher oscillatory shear stress increased NADPH oxidase expression, elevated reactive oxygen species production, and activated the p-AKT/BIRC5 signalling axis, promoting VSMC proliferation, migration, and matrix metalloproteinases 2 and 9 (MMP-2, MMP-9) expression.

### Mechanosensors and the Regulation of EV Release

ECs detect haemodynamic forces through several distinct classes of mechanosensors. These include the mechanically activated ion channel Piezo1, the junctional complex formed by PECAM-1, VE-cadherin and VEGFR2, G-protein-coupled receptors, integrins, primary cilia, and the apical glycocalyx [13].

Among these sensors, Piezo1 has attracted attention as a candidate upstream regulator linking flow sensing to vascular inflammation. Albarrán-Juarez et al. [26] demonstrated in mouse arterial endothelium that both laminar and disturbed flow activate Piezo1, but only disturbed flow leads to integrin-dependent nuclear factor kappa B (NF-κB) activation and the progression of atherosclerosis. In 2016, Wang et al. [27] showed, using bovine aortic ECs and mouse arterial endothelium, that Piezo1 mediates flow-induced ATP release through pannexin channels, activating downstream AKT/eNOS signalling and regulating blood pressure. Lan et al. [28] identified a Piezo1-Ca2+/CaM/CaMKII-FAK/Src-YAP mechanotransduction axis in HUVECs exposed to oscillatory shear stress and showed that the Piezo1 inhibitor GsMTx4 delayed atherosclerotic plaque progression in vivo in ApoE-/- mice.

No published study has directly demonstrated that Piezo1 controls endothelial EV biogenesis, cargo loading, or release. The connection between Piezo1 mechanotransduction and EV regulation we discuss is inferred only from overlapping pathways, as Piezo1 activates the same intracellular cascades, including calcium influx, ERK1/2, Rho GTPases, and NF-κB that independently regulate EV secretion through multivesicular body (MVB) fusion and membrane budding [29,30]. Calcium influx through Piezo1 could plausibly promote MVB–plasma membrane fusion events that release exosomes, while Rho-mediated cytoskeletal remodelling could facilitate microvesicle shedding. However, this is hypothetical and requires direct experimental confirmation in long SV and venous ECs exposed to defined flow patterns. Given that vein grafts experience predominantly disturbed flow in the early post-implantation period (days to weeks), if Piezo1-mediated signalling does regulate EV production and cargo, this would be expected to generate EVs enriched with pro-inflammatory and pro-proliferative molecules. Beyond ECs, Piezo1 is also expressed in VSMCs and is upregulated in human and mouse neointima. Zhang et al. demonstrated that VSMC-specific Piezo1 knockout blocks disturbed-flow-induced proliferation, migration and synthetic phenotype switching via a Ca2+/CaMKII-calcineurin-YAP/TAZ axis [31]. While this study did not examine EV release, it establishes Piezo1 as a mechanosensor in the cell type most directly responsible for IH. Separately, Abello et al. showed that endothelial Piezo1 regulates VSMC differentiation in large arteries [32], suggesting that Piezo1-mediated EC–VSMC crosstalk, potentially via EVs, warrants investigation in the context of vein grafts.

## 3. Cell Specific EV Contributions to Vein Graft Disease

In comparison to our context of studying EVs in VGD, the role of EVs in native vessel atherosclerosis is well established. Endothelial-, smooth muscle-, platelet- and macrophage-derived EVs each contribute to lesion initiation, progression and calcification through the cargo transfer of microRNAs, cytokines and procoagulant lipids [33,34,35,36,37,38,39,40]. Both the diversity of cellular EV sources in atherosclerosis and the role of EV-associated non-coding RNAs across cardiovascular disease have been the subject of recent comprehensive reviews [41,42]. VGD shares several of these effector cells with atherosclerosis but is primarily distinguished in pathology by the abrupt mechanical transition at implantation, surgical denudation injury, and by a compressed timeline in which IH, rather than lipid-driven plaque formation, dominates in the early phase [5,20]. Table 1 summarises the evidence for each cell type’s contribution in this specific context, and where possible we distinguish findings derived from arterial atherosclerosis from those (albeit limited) that have been confirmed in venous tissue.

### 3.1. Endothelial Cell-Derived EVs

Endothelial cells (ECs) are the first vascular cell type to encounter altered haemodynamic forces after vein graft implantation, and the EVs they release carry miRNA cargo that directly influences the behaviour of neighbouring VSMCs, monocytes, and other ECs. EC-derived EVs influence vein graft pathophysiology through their miRNA cargo and surface molecule expression. miR-126 is the most abundant endothelial-enriched miRNA and suppresses two targets, both validated in human EC lines and zebrafish vasculature: PIK3R2, a negative regulator of VEGF signalling, and SPRED1, a suppressor of Ras/MAPK signalling. By targeting these molecules, miR-126 promotes pro-angiogenic and pro-survival signalling in recipient cells [33,34,35]. Arderiu et al. [36] used activated HUVECs to demonstrate that tissue factor (TF) -rich endothelial microvesicles carry high miR-126 levels proportional to TF expression. These microvesicles induced monocyte differentiation into endothelial-like cells through SPRED1/PIK3R2 targeting. This mechanism has direct implications for the re-endothelialisation of vein grafts, where re-endothelialisation is required following denudation of the venous endothelium during surgical harvest. Without re-endothelialisation, the exposed subendothelial matrix drives acute thrombosis and initiates IH [56]. EC-derived EVs may also contribute to vascular calcification in the diabetic setting. Lin et al. demonstrated that high-glucose-stimulated EC-derived EVs induce VSMC calcification via a circ_0008362/miR-1251-5p/Runx2 axis, with clinical correlation between EV circ_0008362 levels and coronary and aortic calcification severity in diabetic patients [43]. Given the high prevalence of diabetes in CABG cohorts, EC-derived EV cargo may differ in diabetic patients in ways that accelerate calcific VGD.

Endothelial EVs also carry miR-143/145, a second miRNA cluster with direct relevance to VSMC proliferation in vein grafts. Two independent studies have examined miR-143/145 transfer between ECs and VSMCs, but they describe transport in opposite directions, mediated by different delivery mechanisms and triggered by distinct stimuli. Hergenreider et al. [12] demonstrated that laminar shear stress activates KLF2 in HUVECs, which package miR-143/145 into EVs and release them to co-cultured human aortic VSMCs (arterial origin), suppressing their proliferative capacity. The direction of transfer here is EC-to-VSMC, and the vehicle is EVs. However, Climent et al. [14] showed that when TGF-β signalling is active, human coronary artery SMCs transfer miR-143/145 back to HUVECs via tunnelling nanotubes rather than EVs. In this case, the direction is reversed (VSMC-to-EC) and the vehicle is a direct physical bridge between cells rather than a secreted particle. The same cargo therefore travels in opposite directions depending on the local stimulus. Flow drives EC-to-VSMC transfer via EVs whereas TGF-β drives VSMC-to-EC transfer via nanotubes. In vein grafts, where disturbed flow and elevated TGF-β coexist, the dominant transfer direction may shift across the remodelling timeline. Interventions targeting miR-143/145 delivery may therefore need to account for the phase of graft adaptation. Endothelial microparticles may also serve as biomarkers of endothelial dysfunction. Koga et al. [57] showed, using flow cytometry on human plasma, that CD144+ endothelial microparticles correlate inversely with coronary endothelium-dependent vasodilation in patients with type 2 diabetes and CAD. This inverse relationship has implications for vein graft surveillance, as endothelial dysfunction is one of the earliest detectable events in VGD progression and this inverse relationship suggests CD144+ microparticles could serve as a form of surveillance in this setting [11]. No study has characterised miR-126 or miR-143/145 packaging into EVs from human SV-derived ECs. All evidence described above derives from HUVECs, human coronary artery smooth muscle cells, ApoE knockout mouse aortas, or a rabbit venous interposition graft model.

### 3.2. VSMC-Derived EVs

VSMC-derived EVs contribute to IH and vascular calcification, two key features of VGD. Calcifying matrix vesicles, enriched with tissue-nonspecific alkaline phosphatase and phosphatidylserine, range from approximately 30 to 520 nm and represent a direct pathogenic mechanism in late-stage disease [37,58]. Key regulators of calcifying EV biogenesis include caveolin-1 in human aortic VSMCs (arterial origin), where EGFR inhibition prevents calcifying EV production [46], and sortilin in human coronary artery SMCs and ApoE-/- mouse aortas (arterial tissue), which directs alkaline phosphatase loading into calcifying EVs [45].

Circulating small EVs also promote VSMC dysfunction through receptor tyrosine kinase signalling. Lee et al. [59] demonstrated that circulating serum-derived small EVs at physiological concentrations dose dependently increased VSMC migration by two-fold and promoted synthetic phenotype markers in human aortic VSMCs, whereas inhibition of the AXL and MerTK receptors blocked these effects. These were circulating EVs acting on VSMCs rather than VSMC-derived EVs. This distinction matters for attributing the source of pathological signalling. miR-145 critically regulates VSMC phenotype switching [60]. Cordes et al. [44] demonstrated in multipotent mouse cardiac progenitors and arterial VSMCs that miR-145 directly targets KLF4, elevating contractile protein expression whilst suppressing the proliferative phenotype. A reduction in miR-145 packaging into VSMC-derived EVs which is distinct from a fall in total cellular miR-145 expression or in overall EV release, would be expected to weaken the feedback loop that miR-145 normally imposes on neighbouring VSMC proliferation. This loss could establish a positive feedback loop that amplifies IH in vein grafts. The mechanism has not been tested directly in venous tissue, and the relative contribution of altered cargo loading versus altered EV output remains unknown.

### 3.3. Platelet-Derived EVs

Platelet microparticles constitute 70 to 90% of circulating microparticles. Sinauridze et al. [38] demonstrated that platelet microparticle membranes have 50- to 100-fold higher specific procoagulant activity per unit surface area than activated platelet surfaces in human activated platelets. This amplification of coagulant activity makes platelet EVs particularly relevant to thrombosis, which is seen in acute vein graft failure occurring early, within hours to days, after implantation during CABG. Beyond thrombosis, platelet EVs drive IH through direct effects on VSMCs. Vajen et al. [47] confirmed that platelet EVs induce proliferation through CD40-CD40L and P-selectin interactions in aortic VSMCs and that they drive migration through CXCL4 presented on the EV surface. Liu et al. [48] used a rat carotid artery balloon injury model to show that platelet-derived microvesicles promote VSMC de-differentiation via the Src/Lamtor1/mTORC1 pathway, suppressing contractile markers. SMC-specific Lamtor1 knockout in mice showed attenuated IH. No equivalent studies have been performed in venous tissue.

miR-223 is the most abundant platelet miRNA and acts as a counter-regulatory brake. Laffont et al. [49] demonstrated, using HUVECs, that activated human platelets deliver functional Ago2-miR-223 complexes via microparticles, resulting in a 22-fold increase in HUVEC miR-223 levels that is sustained for 48 h. Zeng et al. [50] demonstrated in vivo uptake of platelets by VSMCs at femoral artery wire-injury sites, showing that miR-223 directly targets PDGFRβ to suppress VSMC proliferation. miR-223 knockout mice showed increased IH, while agomiR-223 delivery reduced neointimal formation. Su et al. [51] used diabetic mice to show that after femoral artery injury, diabetic platelets are deficient in miR-223 due to calpain-mediated degradation, and restoring miR-223 via calpeptin attenuated IH by suppressing the IGF-1R/AMPK axis. These findings suggest that platelet-derived miR-223 acts as a protective mechanism that interrupts IH, a process that fails in certain physiologies, such as diabetes. Whether this mechanism operates equivalently in venous smooth muscle cells remains to be determined. Platelet EV biology is not tissue-specific in the same way as endothelial or VSMC EV biology, because platelets circulate rather than reside in the vessel wall. However, no study has measured platelet EV deposition, uptake or miR-223 delivery specifically at a vein graft surface or in venous smooth muscle cells.

### 3.4. Macrophage, Foam Cell and Neutrophil Derived EVs

Neutrophil microvesicles drive atherosclerosis progression by delivering miR-155 to atheroprone arterial endothelium, increasing NF-κB activation and plaque macrophage content, as shown in ApoE-/- mice and human arterial EC cultures [39]. miR-155 disrupts endothelial barrier integrity by targeting tight junction proteins ZO-1 and Claudin-1, potentially facilitating inflammatory cell infiltration into the vein graft wall by disrupting endothelial barrier function [52]. Niu et al. [40] showed that foam cell-derived EVs promote VSMC migration and adhesion through the ERK and Akt pathways, using human THP-1-derived foam cells and human aortic VSMCs. Bouchareychas et al. [53] demonstrated in ApoE-/- mice (arterial tissue) that macrophage-derived exosomes carrying miR-99a, miR-146b, and miR-378a reduced aortic lesion macrophage content and necrotic area, suggesting they may have therapeutic value if they have the same effect in arterialized venous tissue. Yang et al. provided further evidence for the pro-atherogenic role of exosomal miR-155 [54]. They demonstrated that plasma exosomal miR-155-5p captures lipopolysaccharide, drives foam cell formation by targeting DET1, and accelerates atherosclerosis in ApoE knockout mice [54]. Not all macrophage-derived EV effects are pro-atherogenic. Bai et al. demonstrated that macrophage-derived exosomes carrying miR-204-5p suppress VSMC calcification by targeting RUNX2 via AT2 receptor activation [55]. This protective mechanism suggests that macrophage EV biology in the vein graft wall may include both pathological and reparative signalling, depending on the macrophage polarisation state and the local microenvironment. No study has evaluated macrophage-derived EV effects specifically in venous tissue or vein graft models.

## 4. EV-Associated miRNA Biomarkers

Several EV-associated microRNAs have established roles in native atherosclerosis. miR-21, miR-126, miR-223 and miR-155 each regulate endothelial activation, smooth muscle phenotype, platelet function or macrophage polarisation in arterial disease, and their circulating levels track with disease activity in coronary and peripheral arterial cohorts [61,62,63,64,65]. Their utility as proposed vein graft biomarkers within this review is based on the assumption that the same regulatory circuits operate in arterialised venous tissue. We also assume that a graft-specific signal can be extracted against the background of native arterial disease that is present in 100% of CABG patients undergoing surgery for obstructive CAD. We review each candidate in turn before considering the standardisation and confounding issues that currently prevent clinical translation. A summary of the proposed biomarkers is also reported in Table 2. 

Multiple EV-associated miRNAs show biomarker potential in cardiovascular populations though none have been validated specifically for VGD diagnosis or surveillance (Table 2). miR-21 is the most extensively validated. A meta-analysis by Wang et al. [61] evaluating circulating miR-21 as a diagnostic biomarker for acute myocardial infarction (MI) reported a pooled sensitivity of 0.83, specificity of 0.81, and a weighted area under the summary receiver operating characteristic curve of 0.85 (all with 95% confidence intervals below 1). Key targets include PTEN, Smad7, and PDCD4. Given its role in fibrotic signalling and the prominence of extracellular matrix deposition in IH, miR-21 warrants evaluation as a biomarker of VGD.

A central difficulty for an EV-miRNA biomarker proposed for vein graft surveillance is biological non-specificity. miR-21, miR-126, miR-223 and miR-155 are all pleiotropic and dysregulated in conditions like diabetes, chronic kidney disease, heart failure and atrial fibrillation which are all highly prevalent conditions in patients undergoing CABG [61,62,63,64]. The peri-operative environment also adds additional confounding features; cardiopulmonary bypass, ischaemia–reperfusion injury, the transfusion of stored blood products, anaesthetic agents and systemic inflammatory responses all transiently alter circulating EV concentrations and miRNA cargo in the first days after CABG [66,67]. These observations are supported by a randomised study comparing on-pump and off-pump CABG which showed that cardiopulmonary bypass produces a more pronounced rise in platelet-, endothelial- and B-cell-derived EVs than off-pump surgery, with changes correlating to pro-inflammatory cytokine profiles [68]. Sampling protocols therefore intended to capture a graft-related or derived signal must define the timing of any collections with reference to these confounders and ideally include a pre-operative baseline to allow patients to act as autologous controls, and a delayed sample after the acute peri-operative response has resolved.

miR-126 is the most abundant endothelial miRNA and has already been discussed in the context of EC-derived EVs above. Its relevance as a biomarker stems from its EC specificity and its responsiveness to vascular injury. Circulating EV-associated miR-126 is reduced in diabetic stroke patients and elevated within 48 h in acute myocardial infarction [65]. High glucose [62] conditions reduce miR-126 content in EC-derived EVs, suggesting that metabolic stress alters endothelial EV cargo. In the vein graft setting, where endothelial denudation during harvest is a primary initiating event [56], changes in circulating EV-associated miR-126 could reflect the degree of endothelial recovery or ongoing dysfunction within the graft.

miR-223 has been discussed above as the most abundant platelet miRNA and as a protective brake on VSMC proliferation via the targeting of PDGFRβ and IGF-1R [49,50,51]. As a biomarker, circulating miR-223 is inversely associated with major adverse cardiovascular events and serves as an independent predictor of high-on-treatment platelet reactivity [63]. Given that platelet activation drives both early thrombotic graft occlusion and the initiation of IH, EV-associated miR-223 levels may provide simultaneous information about thrombotic risk and the inflammatory status of a graft. Whether miR-223 levels change in a predictable pattern after CABG has not been studied.

**Figure 2:**
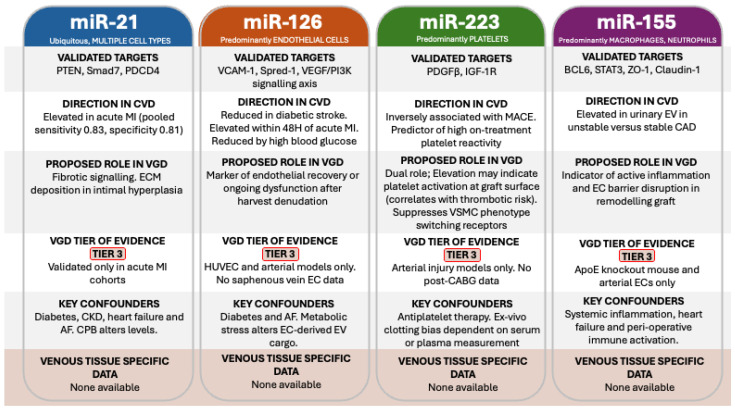
EV-associated miRNA biomarker candidates for VGD surveillance.

miR-155 is elevated in urinary EVs from patients with unstable compared with stable CAD [64] and its validated targets include BCL6, STAT3, and the tight junction proteins ZO-1 and Claudin-1 [52]. Given miR-155s role in promoting endothelial barrier disruption and inflammatory cell infiltration, as discussed in the macrophage EV section above, EV-associated miR-155 could serve as an indicator of active inflammatory processes within a remodelling vein graft. Hypothesised mi-RNA candidate biomarkers for the surveillance of VGD are summarised in Figure 2.

Standardisation challenges currently prevent the clinical implementation of any of these miRNA biomarkers [8]. Pre-analytical variables, including the timing of collection, the choice between serum and plasma, haemolysis, and concomitant medications, all influence EV isolation and characterisation. The EV isolation method is a major pre-analytical variable. Differential ultracentrifugation is the most widely used technique but co-isolates lipoprotein particles and protein aggregates that contribute substantially to the apparent miRNA pool especially within plasma [69]. Size exclusion chromatography improves purity but at the cost of yield and is therefore not well-suited to high-throughput clinical workflows [70]. Polymer-based precipitation methods are convenient but co-precipitate non-vesicular contaminants that confound downstream miRNA quantification [71]. The choice between serum and plasma also matters because clotting releases platelet-derived EVs and platelet-resident miRNAs, including miR-223, into serum at concentrations that obscure the circulating signal [72]. Haemolysis of blood samples for testing can also be problematic because it releases miR-451a and miR-16 from red blood cells which are commonly used as reference genes and so a small degree of haemolysis can shift the apparent expression of targets by an order of magnitude [73].

Analytical variability is equally challenging. There is no validated endogenous reference miRNA for normalising EV-associated small RNAs and most studies use either spike-in controls such as cel-miR-39 or geometric means of stable transcripts but neither of these approaches have been validated in CABG cohorts [8,73]. The quantitative platform used can also influence results. Reverse transcription quantitative PCR, small RNA sequencing and digital droplet PCR each have varied limits of detection, dynamic ranges and biases against short GC-rich sequences and so absolute concentrations are not necessarily comparable and are not interchangeable across platforms [8]. Consensus on isolation methods of choice, sample handling, the normalisation strategy and the best quantification platform is therefore a pre-requisite for comparing between studies and identifying a meaningful clinical translational argument.

## 5. Therapeutic EV Modulation Strategies

Having considered EV-associated microRNAs as candidate biomarkers for vein graft surveillance, we also evaluate therapeutic strategies that may modulate EV signalling itself (Table 3). There are several pharmacological agents already used routinely after CABG which can influence EV release as a secondary effect. There are additional emerging tools designed to target EV biogenesis directly and primarily. Where the agent acts on a specific biogenesis pathway, we note the EV subtype affected.

Key therapeutic strategies targeting EV signalling pathways relevant to VGD are summarised in Table 3. KCa3.1 is upregulated in ECs, VSMCs, macrophages and T lymphocytes in ischaemic heart disease, where it promotes smooth muscle proliferation, neointimal formation and chronic vascular inflammation, and pharmacological blockade reduces atherosclerotic plaque burden and restenosis in animal models [75]. TRAM-34 inhibits KCa3.1-mediated exosome secretion via the AKT/Rab27a signalling pathway [16]. This mechanism is subtype restricted. Rab27a regulates the docking and fusion of multivesicular bodies with the plasma membrane and therefore controls exosome release specifically [76]. Microvesicles formed by outward plasma membrane budding, and platelet microparticles released during platelet activation are not generated through this pathway [7] and would not be expected to respond to KCa3.1 inhibition [16,17]. The therapeutic relevance of TRAM-34 to VGD therefore depends on the relative contribution of exosomes, as opposed to microvesicles and microparticles, to neointimal signalling within an arterialised graft. This contribution is currently unknown and would be an important question for any future evaluation of KCa3.1 inhibition in this setting.

KCa3.1 is upregulated in synthetic phenotype VSMCs, the predominant phenotype in IH. In a swine coronary artery restenosis model, TRAM-34 delivered via balloon catheter prevented SMC phenotypic modulation and limited stenosis [17]. In a rat carotid angioplasty model, Köhler et al. [18] demonstrated that six weeks of TRAM-34 treatment reduced neointimal hyperplasia by approximately 40%. No study has tested KCa3.1 inhibition in SV graft models. However, genetic evidence from a mouse model now complements the available pharmacological data. Alam et al. demonstrated that KCa3.1 silencing in ApoE knockout mice reduced brachiocephalic plaque size and stenosis by approximately 70% with reduced macrophage infiltration [77]. They also showed that KCa3.1 inhibition suppresses PDGF-BB-induced VSMC MCP-1/Ccl2 expression in vitro [77]. This genetic loss-of-function evidence strengthens the case for KCa3.1 as a viable therapeutic target, though its efficacy in venous tissue remains untested.

Antiplatelet agents commonly used after CABG surgery differentially affect platelet EV release. Cheng et al. [78] measured circulating microparticle concentrations in patients with stable angina compared with healthy controls and found that EV levels were significantly elevated (5.76 ± 0.86 mg/mL versus 2.62 ± 0.49 mg/mL, *p* < 0.05), with platelet-derived MPs (CD31+/CD41+) accounting for approximately 36% of the total. Using rat thoracic aortas incubated with these patient-derived MPs, they demonstrated decreased ERK1/2 expression, increased JNK and p38 MAPK phosphorylation, elevated NF-κB and VCAM-1 levels, reduced nitric oxide production, and increased superoxide generation. Following one week of aspirin treatment, total EV concentrations fell significantly (3.96 ± 0.73 mg/mL, *p* < 0.05), with platelet-derived MPs showing the most pronounced reduction. Aspirin also attenuated the pro-inflammatory and pro-oxidant effects of EVs on aortic tissue. Gasecka et al. [79] compared other commonly used post-operative antiplatelet agents, ticagrelor and clopidogrel, in humans following acute MI and found that platelet EV concentrations were significantly lower on ticagrelor at discharge and at six months. Given that platelet EVs drive both thrombosis and IH, the choice of antiplatelet regimen after CABG may influence EV-mediated signalling within vein grafts.

MSC-derived EVs demonstrate efficacy in preclinical vein graft models. Qu et al. [74] administered human umbilical cord MSC exosomes in a rat jugular vein-to-carotid artery graft model and achieved a 40% reduction in neointimal thickness (84 ± 27.69 μm versus 149.7 ± 38.58 μm, *p* < 0.05), with preserved luminal diameter and reduced proliferating cells. Mechanisms included accelerated re-endothelialisation, increased eNOS expression, and reduced MMP-2/9 activity via PI3K/AKT and MAPK/ERK1/2 signalling. This is among the few studies directly evaluating EV therapy in a vein graft model and provides a proof of concept for clinical translation. There is also an emerging translational approach combining EV biology with device engineering. Xiao et al. coated vascular stents with dual endothelial-cell- and MSC-derived exosomes, demonstrating promoted re-endothelialisation, suppressed macrophage adhesion, induced VSMC contractile-phenotype switching, and reduced neointimal thickness [80]. This concept could be adapted for external vein graft stenting, where the local delivery of pro-reparative EV cargo to the graft adventitia at the time of implantation could be evaluated.

Statins modulate endothelial EV release. Tramontano et al. [81] demonstrated, using TNF-α-activated human coronary artery ECs, that fluvastatin suppresses endothelial microparticle release by inhibiting the Rho/Rho-kinase pathway. Given that statins are routinely prescribed after CABG, their EV-modulating effects may contribute to graft protection independently of their cholesterol-lowering effects.

## 6. Prognostic Evidence and the Vein Graft Evidence Gap

EVs already demonstrate prognostic value in several cardiovascular populations. No studied population is directly comparable to the vein-specific CABG setting, but parallels can be drawn from conditions that share features of endothelial injury and abrupt haemodynamic loading, and we draw these parallels with explicit caveats below. Amabile et al. [82] showed in 81 patients with end-stage renal disease that endothelial EV levels independently predicted cardiovascular-specific mortality. End-stage renal disease produces sustained endothelial activation through uraemic toxins, oxidative stress, and haemodynamic load and has some shared features with the arterialized venous endothelium in a SV graft where an abrupt step-change in mechanical stress drives acute endothelial injury. Whether EV subtypes produced under chronic uraemic stress react similarly to those generated by acute surgical trauma and arterial haemodynamic conditioning remains untested.

Nozaki et al. [83] demonstrated in 169 patients with heart failure that CD144+ endothelial EV levels increased with New York Heart Association breathlessness class and independently predicted cardiovascular events (HR 2.423, 95% CI: 1.034–5.681, *p* = 0.04). Heart failure involves reduced cardiac output and venous congestion, a haemodynamic profile distinct from the high-shear, high-pressure environment a vein graft experiences after CABG. CD144 marks endothelial adherens junction disruption, which is relevant to graft biology given that surgical handling and distension injury compromise junction integrity acutely, but the chronic low-output stimulus driving EV release in heart failure differs mechanistically from surgical denudation.

Sinning et al. [84] showed that CD31+/Annexin V+ microparticles independently predicted cardiovascular events in 200 stable CAD patients followed for 6.1 years. Stable CAD patients carry persistent low-grade endothelial activation over atherosclerotic plaques. Vein graft failure, particularly in the early and intermediate phases, is driven instead by neointimal hyperplasia and thrombosis rather than plaque rupture, so the biological stimulus generating EVs in these two contexts differs, even if the EV surface markers partially overlap.

The closest available evidence linking EVs to graft outcomes comes from Camera et al. [19] who, in a nested case–control sub-study of the CAGE trial, performed flow cytometry on plasma from 60 of 179 CABG patients who underwent CT angiography at 18 months, reporting a 24% graft occlusion rate. Patients with occluded grafts had two-fold higher activated platelet-derived EVs (CD41+/CD62P+) and four-fold higher TF-positive EVs at baseline, on the day before surgery. Elevated platelet activation markers and TF loading before surgery are plausible surrogates for a prothrombotic phenotype that is directly relevant to early graft occlusion, given that thrombosis within the first month drives a substantial proportion of early failures. A cumulative scoring system achieved an AUC of 0.897 (95% CI: 0.81–0.98, *p* < 0.0001) for predicting graft occlusion. However, this study examined all grafts (both arterial and venous) collectively without isolating SV grafts. Arterial and venous conduits fail through different mechanisms and at different rates [3]. Baseline sampling also precludes any assessment of how the EV phenotype evolves in response to surgery, ischaemia–reperfusion injury or haemodynamic loading as seen during CABG.

No prospective study has collected serial EV data from CABG patients and linked EV phenotypes to graft patency outcomes. VGD has a distinct pathophysiology that differs from that of native arterial vessel atherosclerosis and is driven by mechanical stress and surgical injury. CABG patients constitute a well-defined cohort with a known time-zero event, thereby facilitating prospective longitudinal biomarker studies. Current methods for monitoring vein graft patency are impractical for routine surveillance because they require exposing the patient to ionising radiation. Therefore, a blood-based EV biomarker could address this clinical need. Although no study has yet linked EV phenotypes to graft patency specifically, prospective EV data from CABG patients are now emerging for other post-operative endpoints. Liu et al. demonstrated in a multi-centre prospective discovery and validation CABG cohort that EVs released in pericardial drainage fluid carry profibrotic miR-4324 and predict post-operative atrial fibrillation via the SKP1-TGF-β1/Smad3 axis [85]. Separately, Urbanowicz et al. showed that leukocyte-derived EV counts in plasma vary by graft type in off-pump CABG patients [86]. Both studies support the feasibility of prospective EV phenotyping in this surgical population. Analogous designs could be applied to the question addressed in this review about vein graft patency.

## 7. Conclusions

This review summarises mechanistic evidence linking EVs to each phase of VGD, from acute mechanotransduction-triggered EV release upon arterial implantation through to endothelial dysfunction and VSMC phenotypic switching, as well as chronic inflammation and calcification. A key limitation is that most cited evidence derives from arterial tissue models or non-vascular cell types. A small number of studies have used venous cells [15], venous graft models [74], or human SVs [19], but the remaining evidence base is inferred solely by extrapolation from arterial biology. Venous and arterial vascular cells differ in their contractile phenotype, in baseline expression of shear-responsive transcription factors and in the proliferative response to vascular injury. Whether EV biogenesis, cargo loading, release kinetics and recipient cell uptake operate equivalently in arterialised venous tissue is currently unknown. The conclusions of this review should be considered as hypothesis-generating for the vein graft setting and not as an established or proven mechanism. Addressing these gaps through studies using human SV tissue and prospective CABG cohorts will be essential for translating the substantial preclinical evidence base into clinical benefit. Prospective studies examining EV profiles at defined time points following CABG and correlating these with graft patency outcomes are urgently needed before it can be determined whether these are suitable as biomarkers or therapeutic targets.

## Figures and Tables

**Table 1:** Cell specific extracellular vesicle contributions to vein graft disease.

Cell Type or EV Source	Key EV Cargo or Surface Features	Primary Recipient Cells	VGD Relevant Effects	Key Pathways or Mediators	Key Evidence Cited	Vein Graft Specific Evidence Strength
Endothelial cell-derived EVs	miR-126, miR-143/145, tissue factor enriched EVs, circ_0008362	VSMCs, monocytes, endothelial cells	Re-endothelialisation, endothelial dysfunction signalling, modulation of VSMC proliferation, VSMC calcification in diabetes	SPRED1, PIK3R2, KLF2 shear response, VEGF signalling, circ_0008362/miR-1251-5p/Runx2	Hergenreider 2012 [12]; Arderiu 2022 [36]; Harris 2008 [33]; Fish 2008 [34]; Wang 2008 [35]; Lin 2024 [43]	Indirect, extrapolated from endothelial models
VSMC-derived EVs	Calcifying matrix vesicles, phenotype associated cargo, miR-145 context	Adjacent VSMCs, extracellular matrix	Phenotypic switching, intimal hyperplasia progression, vascular calcification	Sortilin, caveolin-1, EGFR, KLF4 regulation via miR-145	Cordes 2009 [44]; Kapustin 2015 [37]; Goettsch 2016 [45]; Bakhshian Nik 2023 [46]	Indirect, mainly arterial VSMC models
Platelet-derived EVs	High procoagulant activity, CD40L, P-selectin, CXCL4, miR-223	Endothelium, VSMCs	Early thrombosis, VSMC proliferation, migration, de-differentiation	Coagulation amplification; Src Lamtor1 mTORC1; PDGFRβ targeting by miR-223	Sinauridze 2007 [38]; Vajen 2017 [47]; Liu 2021 [48]; Laffont 2013 [49]; Zeng 2019 [50]; Su 2020 [51]	Mechanistically strong, limited SVG specific data
Macrophage or foam cell-derived EVs	miR-155; miR-99a, miR-146b, miR-378a, miR-155-5p, miR-204-5p	VSMCs, endothelium	Inflammation, barrier disruption, VSMC migration and adhesion, resolution signalling, LPS capture, foam cell formation, suppression of VSMC calficiation (protective)	NF-κB activation; tight junction disruption; ERK and AKT signalling; DET1 targeting; Runx2 targeting via AT2R	Gomez 2020 [39]; Pena-Philippides 2018 [52]; Niu 2016 [40]; Bouchareychas 2020 [53], Yang 2024 [54], Bai 2025 [55]	Indirect, no dedicated SVG studies
Mechanical forces shaping EV biology	Shear-dependent EV cargo shifts and EV uptake via MCAM/PECAM-1, increased EV release	Multiple vascular and immune cells	Amplified early injury signalling after arterialisation, macrophage inflammatory polarisation	Oxidative stress; YAP mechanosensing; KLF2 laminar signalling; NADPH oxidase axis; MCAM/PECAM-1 adhesion mediated uptake; MAPK pathway	Qin 2022 [23]; Guo 2021 [24]; Hergenreider 2012 [12]; Yao 2023 [15]; Coly 2024 [22]; Hou 2025 [25]	No direct human SVG mechanotransduction EV data

**Table 2:** EV-associated miRNAs and EV modulation strategies.

Proposed Bio-Marker	Proposed Relevance to VGD	Mechanistic Rationale	Key Evidence Cited	SVG Translation Status
miR-21	Marker of remodelling and fibrosis	Fibrotic signalling and matrix remodelling relevant to intimal hyperplasia	Wang 2024 [61]	Not validated for SVG prediction
miR-126	Marker of endothelial injury and repair	Endothelial-enriched miRNA responsive to vascular injury and metabolic stress	Harris 2008 [33]; Fish 2008 [34]; Wang 2008 [35]; Yuan 2022 [65]; Siwaponanan 2023 [62]	Not validated for CABG SVG surveillance
miR-223	Marker of platelet activation and anti-proliferative brake	Suppresses VSMC proliferation via PDGFRβ; altered in diabetes	Laffont 2013 [49]; Zeng 2019 [50]; Su 2020 [51]; Shi 2015 [63]	Not studied longitudinally after CABG
miR-155	Marker of inflammatory activation	Promotes endothelial inflammation and tight junction disruption	Gomez 2020 [39]; Pena-Philippides 2018 [52]; Fitzsimons 2020 [64]	No SVG specific validation

**Table 3:** Therapeutic EV modulation strategies relevant to VGD. Only MSC-derived EVs have been tested in a venous graft model [74]. All other agents have evidence from arterial models or non-graft patient cohorts only. Whether EV-modulating effects observed in arterial tissue translate to the arterialised saphenous vein environment is unknown for all agents except MSC-derived EVs. Therefore, any mechanisms in this regard are hypothesised only. Evidence tier: Tier 1 = direct human patient data. Tier 2 = venous cell systems or venous interposition graft non-human animal models. Tier 3 = arterial cell systems and arterial injury models. Abbreviations: COX, cyclooxygenase; EC, endothelial cell; eNOS, endothelial nitric oxide synthase; EV, extracellular vesicle; MI, myocardial infarction; MMP, matrix metalloproteinase; MP, microparticle; MSC, mesenchymal stromal cell; MVB, multivesicular body; SMC, smooth muscle cell; TNF-α, tumour necrosis factor alpha.

Agent	Mechanism of EV Modulation	EV Subtype Affected	Preclinical Model	Key Outcome	Evidence Tier	Venous Tissue Data	Post-CABG Use
**Aspirin** COX inhibitor	Reduces platelet EV release. Attenuates pro-inflammatory and pro-oxidant effects of circulating EVs on vascular tissue.	Platelet microparticles (CD31+/CD41+)	Human plasma from stable angina patients. Ex vivo rat thoracic aorta incubation	Total EV concentrations reduced after 1 week. Platelet-derived MPs showed most pronounced reduction. Pro-inflammatory effects attenuated	**Tier 1 model** EV levels measured in human but non-CABG patients **Tier 3 tissue** Functional effects tested on rat aorta not venous tissue	No Rat aorta	Routine
**Ticagrelor** P2Y12 inhibitor	Reduces platelet EV release more effectively than clopidogrel	Platelet EVs	Human post-MI patients. Randomised comparison with clopidogrel	Platelet EV concentrations significantly lower at discharge and at 6 months compared to clopidogrel	**Tier 1 model** EV levels measured in humans post-MI but not a CABG cohort. No tissue functional studies.	No Post-MI cohort only	Routine
**Fluvastatin** HMG-CoA reductase inhibitor	Supresses endothelial microparticle release by inhibiting Rho/Rho-kinase pathway	Endothelial microparticles	TNFα activated human coronary artery endothelial cells	Reduced endothelial microparticle release. Potential graft protection independent of cholesterol lowering	**Tier 3** In vitro coronary artery ECs only. No human patient or venous cell data.	No Coronary artery ECs	Routine
**Agents under investigation which target EV biogenesis directly**							
TRAM-34 KCa3.1 channel blocker	Inhibits KCa3.1 mediated exosome secretion via AKT/Rab27a pathway. Subtype-restricted affecting exosomes not micro-vesicle or -particle shedding	Exosomes only (Rab27a-dependent MVB fusion)	Swine coronary artery restenosis, local balloon delivery. Rat carotid angioplasty, 6 weeks systemic	Prevented SMC phenotype modulation and limited stenosis. Approximate 40% reduction in neointimal hyperplasia	**Tier 3** Arterial injury models only. No venous graft or cell data.	No Arterial injury only	Not yet in clinical use
MSC-derived EVs Cell therapy (exosome delivery)	Accelerated re-endothelialisation, increased eNOS expression, reduced MMP-2/9 activity via PI3K/AKT and MAPK/ERK1/2 signalling	MSC exosomes (therapeutic cargo delivery)	Rat jugular vein to carotid artery interposition graft	40% reduction in neointimal thickness (84 vs. 150 μm, *p* < 0.05). Preserved luminal diameter. Reduced proliferation	**Tier 2** Tested in a venous interposition graft animal model	Yes Venous graft non-human animal model	Not yet in clinical use

## Data Availability

No new data were created or analyzed in this study.
